# 913. Incidentally Positive Measles Results with a Commercially Available Multiplex Polymerase Chain Reaction Panel for Exanthems — Tennessee, 2022–2023

**DOI:** 10.1093/ofid/ofad500.958

**Published:** 2023-11-27

**Authors:** Christine Thomas, Amanda Hartley, Caitlin N Newhouse, Timothy F Jones, William Schaffner, Mary-Margaret A Fill, John R Dunn

**Affiliations:** CDC / Tennessee Department of Health, Nashville, Tennessee; Tennessee Department of Health, Goodlettsville, Tennessee; Tennessee Department of Health, Goodlettsville, Tennessee; Tennessee Department of Health, Goodlettsville, Tennessee; Vanderbilt University Medical Center, Nashville, Tennessee; Tennessee Department of Health, Goodlettsville, Tennessee; Tennessee Department of Health, Goodlettsville, Tennessee

## Abstract

**Background:**

In January 2023, the Tennessee Department of Health (TDH) received report of a positive measles result from a multiplex polymerase chain reaction (PCR) panel ordered to evaluate a fever and rash in a child. Immediate public health investigation concluded that the positive test was because of recent measles vaccination. Because multiplex PCR tests have not typically included measles, we assessed patient characteristics, provider responses, and measles virus strain when multiplex PCR identified measles to guide public health response in Tennessee.

**Methods:**

We retrospectively identified Tennessee residents who tested positive for measles by 2 multiplex PCR panels, commercially available since 2022, that include 7–9 viruses and Streptococcus pyogenes. We queried the state immunization registry for measles vaccine records for those persons and asked ordering providers about their clinical assessment, test use, and response to the positive result. CDC performed the Measles Vaccine (MeVA) assay (real-time RT-PCR) on available clinical specimens.

**Results:**

Of 296 multiplex PCR tests on Tennessee residents, 7 (2.4%) were positive for measles. Median age was 1 year (range: 1–6 years). All 7 received a measles vaccine within 8–15 days before the test. Among 7 providers, 6 did not suspect measles prior to testing. All ordered testing to evaluate a rash, with or without fever, and none were aware of patient exposure to persons with measles. After the positive result, 2 providers contacted TDH for guidance, 4 attributed the result to recent vaccination, and 1 reported not having received the test result. Three patients tested positive for additional viruses in the panel including enteroviruses (n = 2), human herpesvirus type 6 (n = 2), human herpesvirus type 7 (n = 1), and Epstein-Barr virus (n = 1). MeVA testing of 2 specimens identified measles vaccine strain in 1 specimen and an inconclusive result in the other.

**Conclusion:**

Inclusion of measles in multiplex PCR panels can incidentally identify measles virus in recently vaccinated persons, requiring MeVA testing to confirm vaccine strain. In recently vaccinated persons, medical and public health professionals must carefully consider use and interpretation of measles results in multiplex PCR panels.

**Disclosures:**

**All Authors**: No reported disclosures

